# Effects of Accelerators and Retarders in Early Strength Development of Concrete Based on Low-Temperature-Cured Ordinary Portland and Calcium Sulfoaluminate Cement Blends

**DOI:** 10.3390/ma13071505

**Published:** 2020-03-25

**Authors:** Taegyu Lee, Jaehyun Lee, Hyeonggil Choi

**Affiliations:** 1 Technology Research & Development Institute, Daelim Industrial, Jongno-Gu, Seoul 03152, Korea; ninga777@naver.com (T.L.); archi0528@daum.net (J.L.); 2 School of Architecture, Kyungpook National University, Buk-Gu, Daegu 4566, Korea

**Keywords:** low-temperature condition, ordinary Portland cement, CSA, accelerator, retarder, early strength

## Abstract

In this study, experiments were performed on the applicability of mortars and concretes based on calcium sulfoaluminate (CSA) binders to facilitate the early strength development of ordinary Portland cement (OPC) under low-temperature conditions. An optimum mixture of CSA was evaluated to improve the early strength of OPC, and the effects of accelerators and retarders on this mixture were examined to demonstrate the applicability of the resulting concrete mixture. Furthermore, mixture applicability was validated by producing concrete at the Remicon Batcher plant and performing numerical simulations. As observed, the optimum CSA substitution rate for the realization of early strength was 17% of the total unit binder amount with CaO/SO_3_ and SO_3_/Al_2_O_3_ ratios of 1.9 and 1.25, respectively. Evidently, CSA in combination with Na_2_SO_4_ as an accelerator promoted the early strength of concrete with OPC and secured its constructability using additional retarders to control the quick setting of concrete. Additionally, the activation of initial hydration at low temperatures yielded a compressive strength of 5 MPa/12 h or higher for the resulting concrete mixture.

## 1. Introduction

In view of its reaction with water to produce hydrates, cement undergoes strengthening through condensation followed by curing [1,2,3,4,5]. This initial hydration reaction produces ettringite and the Ca^2+^ ions present in the liquid phase are continuously eluted to increase the production of Ca(OH)_2_ [6]. Consequently, an acceleration period occurs, in which hydration is reactivated from the C–S–H membrane surrounding the alite (C_3_S) and belite (C_2_S) particles, which undergo expansion and destruction. Subsequently, nuclei are produced in the C–S–H phase to accelerate the growth and rapid consumption of Ca(OH)_2_, which actively produces hydrated products. During the deceleration period, the ettringite membrane surrounding the aluminate particles is destroyed to rehydrate C_3_A, and the compressive strength is expressed through the transition to monosulfates, from insufficient quantities of gypsum [2,6].

Ordinary Portland cement (OPC) is advantageous in that it exhibits favorable physical properties and strength due to its stable chemical reactions, thereby leading to a wide applicability. However, its dependence on high temperatures lowers its reactivity in the low temperature region [7], which limits its early strength development when applied to mortars or concretes.

In contrast, calcium sulfoaluminate cements (hauyne minerals, CSA) have a significantly faster hydration rate than OPC [8,9,10,11]; they have received ongoing research attention since their early strength can be achieved upon substitution in OPC at an optimal ratio—due to the high temperatures involved in the initial hydration reaction [12,13,14,15,16,17,18,19,20]. Additionally, it is possible to secure the formation of a passivation film of the reinforcing steel, in addition to a stable volume due to the excellent initial strength in the early stage, thereby allowing a 28-day design standard early strength to be developed [21,22]. Thus, CSA cements have gained popularity for the early strength development of OPC.

Klein and Metha reported that various hydrates can be produced depending on the molar ratios of Al_2_O_3_/SO_3_ and CaO/SO_3_ in the CaO-Al_2_O_3_-SO_3_ system [23], where a high Al_2_O_3_ component exhibits quick-setting and early strength, a high CaO component exhibits expandability, and a high SO_3_ component exhibits high strength characteristics [24]. The key factors for the hydration of CSA cements vary according to the ingredients and composition of the hauyne clinkers, the reactivity of the externally incorporated gypsum, the type of mixture, and the mixing and curing conditions. These CSA compounds are applied to mortars or concretes and contribute to the expandability of the hardened body using the growth pressure when ettringite crystals grow after the formation of hardened tissues. However, if the expanded pressure exceeds the elastic modulus of the hardened body, it often results in the destruction of the hardened body [23,25]. Therefore, to impart functionality to mortars or concretes, it is desirable to design materials with the characteristics of hydrates such as ettringite, along with developing a comprehensive understanding and sufficient review of the aggregates and the interface, the mixing with mortar, and the concrete to be employed. Although various studies have been conducted on the early strength development of mortars and concretes based on OPC, the majority were conducted on mortar, and there were several limitations due to the initial high hydration heat and quick-setting properties of the CSA cements compared to OPC [26,27,28,29].

In this study, experiments are conducted on mortar and concrete to develop early strength concrete binders using OPC and CSA binders at low temperatures. In addition, this study aims to validate the applicability of CSA in concrete by reviewing various factors, such as the optimum binder, accelerator, and retarder through mortar experiments for early strength development.

## 2. Experimental Work

### 2.1. Materials

Table 1 and Table 2 present the physical characteristics of the materials used in this study. As a cement, OPC (3150 kg/m^3^, fineness: 330 m^2^/kg) was used. As binders, fly ash (density: 2140 kg/m^3^, fineness: 396 m^2^/kg), ground granulated blast-furnace Slag (density: 2860 kg/m^3^, fineness: 430 m^2^/kg), and calcium sulfoaluminate (density: 2890 kg/m^3^, fineness: 466 m^2^/kg) were used.

**Figure 1:**
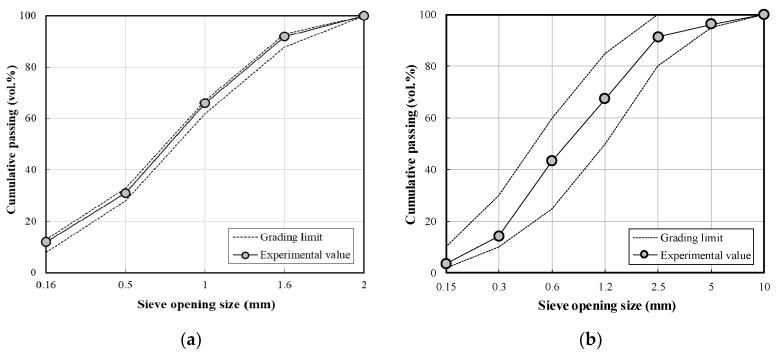

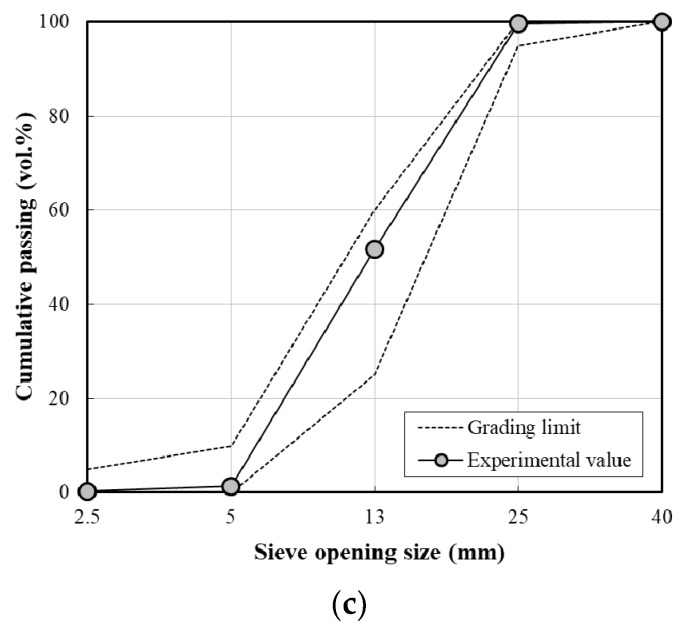
Gradation sieve analysis curves for the aggregates employed herein: (**a**) ISO Standard sand; (**b**) fine aggregates; and (**c**) coarse aggregates.

Figure 1 shows the results of the sieving test of the aggregates (ASTM C136) [30] employed herein, which was carried out to analyze the grading of the fine and course aggregates. For the fine aggregates, ISO standard sand (size: 2 mm, fineness modulus: 2.99, density: 2620 kg/m^3^, SiO_2_: 99%, 0.08 mm, and passage amount: 0.04%) [31] was used in the mortar experiment and sea sand (size: 5 mm, fineness modulus: 2.01, density: 2600 kg/m^3^, and absorption: 0.79%) and crushed sand (fineness modulus: 3.29, density: 2570 kg/m^3^, and absorption: 0.87%) were used to evaluate their applicability in concrete. Fine aggregates were prepared by mixing sea sand and crushed sand in a ratio of 4:6, with a fineness modulus of 2.84.

For the coarse aggregates, crushed granitic aggregates (size: 25 mm, fineness modulus: 6.94, density: 2600 kg/m^3^, and absorption: 0.76%) were used.

The polycarboxylic acid group (density: 1260 kg/m^3^) was used as the chemical admixture. As accelerator materials, NS: Na_2_SO_4_, AS: Al_2_(SO_4_)_3_, CH: Ca(OH)_2_, CN: Ca(NO_3_)_2_, and NC: NaHCO_3_ were used, and as retarders, CA: citric acid, SG: sodium gluconate, BA: boric acid, and TA: tartaric acid were used.

### 2.2. Experimental Outline and Mixture Proportions

Table 3 shows the experimental outline for this study. To examine the optimal method for the early strength development of concrete using ordinary cement, the reactivity of the CSA binder and the amount of additional materials were controlled based on the stoichiometric reaction equations according to CaO/SO_3_ and Al_2_O_3_/SO_3_, and the resulting mortar performance was reviewed [23]. Moreover, the optimal amount of CSA binder that can be substituted for OPC was deduced through mortar performance evaluations based on the amount of CSA binder used.

The possibility of increasing the early strength of mortar by combining a CSA binder and an accelerator was then examined; 0.5% of the total binder was employed as the accelerator. When using CSA binders, quick setting can occur, and so a retarder that is compatible with the CSA binder was reviewed. Five types of retarders were added at a level of 0.2% c.f., the binder. A combination of the accelerator and the retarder was used for preparation of the concrete samples, and the early strength performance and capability to prevent quick setting was reviewed in each case. The retarder quantity was adjusted to 0.2% and the three types of accelerators were used in quantities of 1%, 2%, and 3%.

Finally, a concrete mixing design was formulated based on the amount of CSA, accelerator, and retarder derived from the above experiments. The applicability of early strength concrete binders examined herein was evaluated via mock-up experiments of production at the Remicon Batcher plant.

Table 4 and Table 5 show the mixing proportions of the mortar and concrete used in this study. The flow of mortar was <200 mm according to ASTM C1437 [32]. For the concrete, the slump was 210 ± 25 mm and the air content was 4.5 ± 1.5%.

### 2.3. Test Methods

Table 6 shows the test methods and their corresponding evaluation items for the mortar and concrete samples. The various test procedures are outlined as follows.

#### 2.3.1. Mortar Test

The mortar flow test was conducted based on ASTM C1437 [32], and the mortar setting test was conducted based on ASTM C403/C403M [33]. The compressive strength of the mortar was calculated by measuring the maximum load using 30 ton UTM within the planned time according to ASTM C109/C109M [34]. The compressive strength of the concrete was calculated as an average value obtained from three test specimens.

#### 2.3.2. Application for Concrete

**Figure 2:**
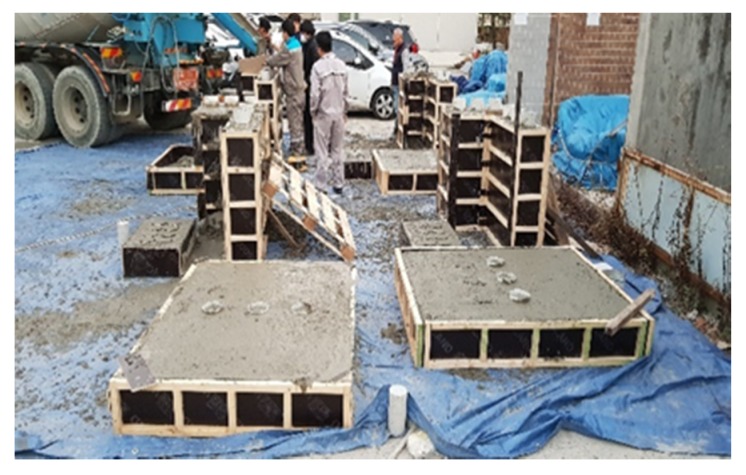
Application test of early strength concrete in the Batcher plant.

**Figure 3:**
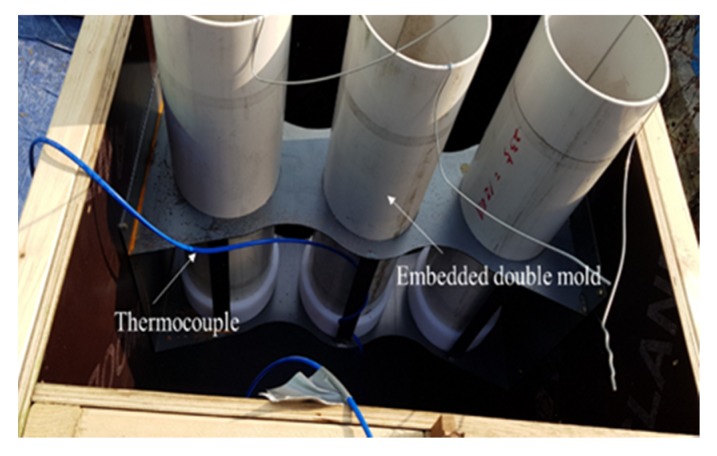
Installation of the thermocouple and embedded double mold for the concrete test.

Based on the ratio of the optimum binder to the admixture derived from the mortar test, the applicability to concrete was evaluated. Concrete was produced at the Remicon Batcher plant, and the hydration heat and strength development were simulated by fabricating mock-up walls. Figure 2 and Figure 3 show the mock-up and thermocouple installation details, respectively. The mock-up simulation was set to 200 mm × 600 mm × 1000 mm and the K-type thermocouple was embedded in the center of the mock-up. 

To evaluate the properties of the fresh concrete, a slump test was conducted based on ASTM C143/C143M [35] and the air content test was conducted based on ASTM C231/C231M-17a [36]. To evaluate the workability at the site, the properties of the fresh concrete were examined after preserving in a mixer truck for 0, 60, and 90 min. To measure the compressive strength of the concrete subjected to hydration, a double mold, as suggested by ASTM C873 [37], was installed.

The compressive strength of the concrete was calculated by first preparing a Ø100 mm × 200 mm specimen according to ASTM C39/C39M [38] and measuring the maximum load using a 300-ton UTM within certain time periods. The compressive strength of the concrete was calculated as the average value obtained from three test specimens.

## 3. Results and Discussion

### 3.1. Properties of the Mortar

#### 3.1.1. Effect of CSA Replacement

**Figure 4:**
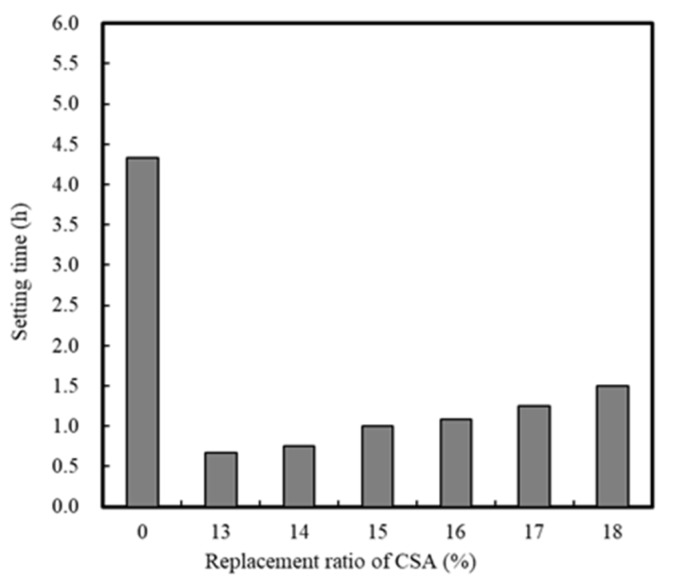
Setting time of mortar with CSA replacement.

**Figure 5:**
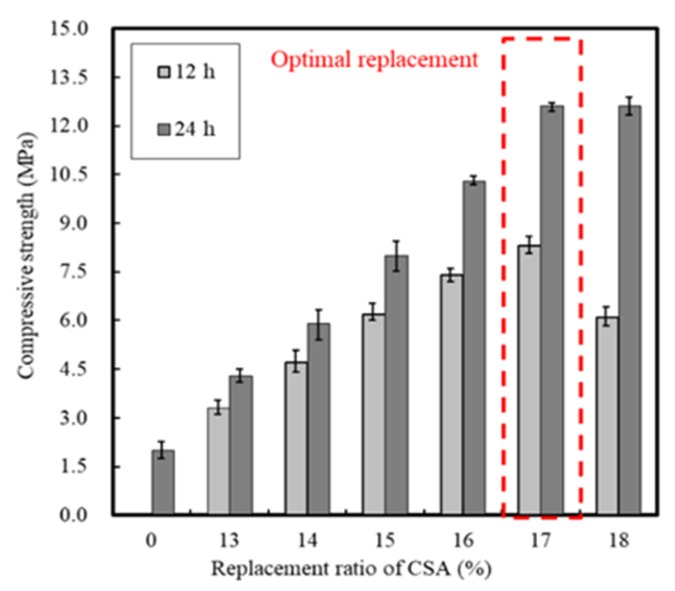
Compressive strength of mortar with CSA replacement.

Figure 4 and Figure 5 show the setting time and compressive strength results, respectively, for the mortar sample with CSA replacement, whereby it is apparent that both increased with an increasing CSA replacement ratio. Above 17% CSA replacement, slight change was observed in the compressive strength, and so this was considered the optimal value. In addition, the compressive strength of mortar reached a maximum at 8.3 MPa, after 12 h, when 17% of CSA was employed in the mixture, and when the setting time was >75 min.

**Figure 6:**
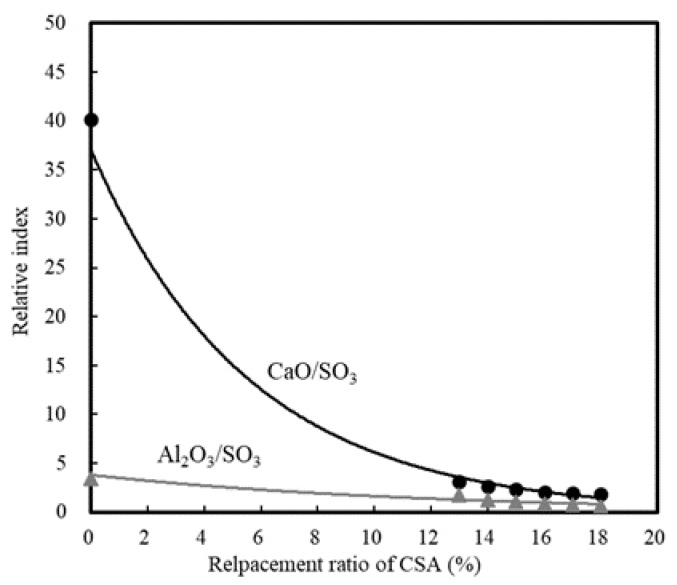
Relative indices of CaO/SO_3_ and Al_2_O_3_/SO_3_ with CSA replacement.

**Figure 7:**
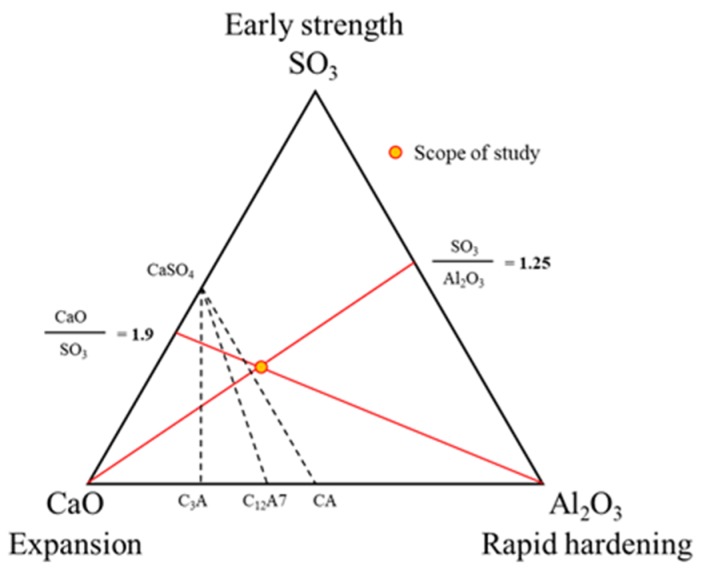
Optimal chemical composition for early strength concrete.

Figure 6 depicts trends in the calculated CaO/SO_3_ and Al_2_O_3_/SO_3_ ratios in accordance with changes in the replacement ratio of CSA, while Figure 7 shows the optimal chemical composition of early strength concrete. Although previous studies confirmed that the chemical properties of CSA contributed to the hydration of cement due to the high content of SO_3_, this effect was only applicable within an appropriate range [39,40,41].

As indicated as a scope of this study, the optimal replacement ratio for the CSA early strength binder equals 17% with CaO/SO_3_ and Al_2_O_3_/SO_3_ ratios of 1.9 and 1.23, respectively.

In addition, it was found that when a combination of the early strength binder, accelerator, and retarder was applied to concrete, a compressive strength of 5 MPa/12 h was realized at low temperatures. However, this also required careful control of the concrete mixing. Moreover, it should be noted that the performances of the early strength binders must be examined under a range of conditions to ensure reliability.

#### 3.1.2. Effect of the Accelerator

**Figure 8:**
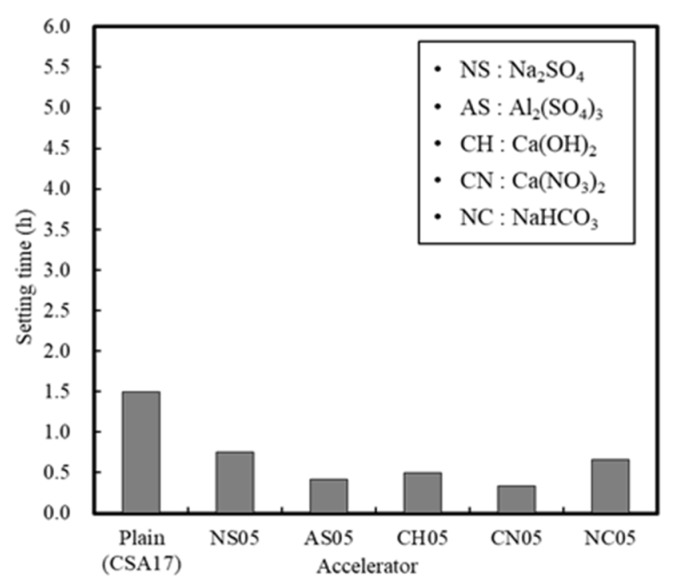
Mortar setting times in the presence of different accelerators.

**Figure 9:**
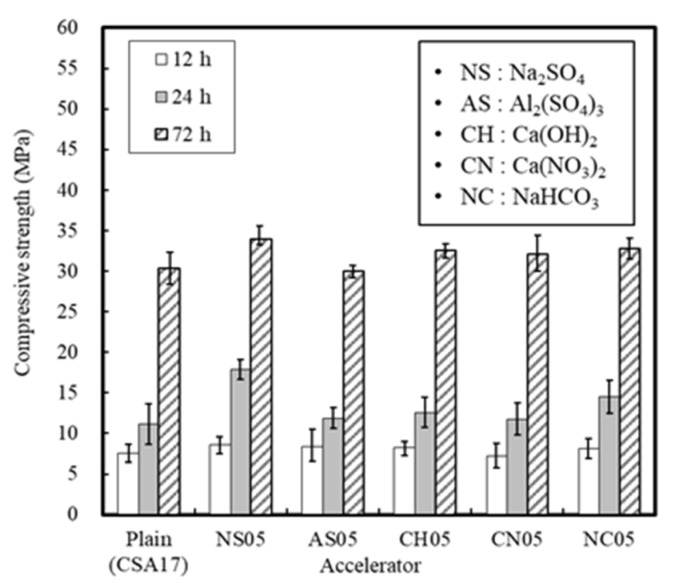
Differences in mortar compressive strengths in the presence of different accelerators.

Figure 8 and Figure 9 show the setting time and compressive strength results for the mortar with added accelerators. In the absence of an accelerator, the setting time of mortar was 90 min, and this rapidly decreased to 20–40 min in the presence of an accelerator. These results indicate that the use of an accelerator promotes the hydration of the reactive hauyne minerals, and since the compressive strength was also affected, it was considered that the initial production of ettringite also increased [19,20].

In particular, Na_2_SO_4_ and Al_2_(SO_4_)_3_ sulfate accelerators were the most effective in improving the initial compressive strength due to their faster ionization rates compared to that of cement, and also because the initially eluted SO_4_^2−^ ions are advantageous for the nucleation of ettringite hydrate. However, although the early strength of mortar can be improved by accelerators, the use of a single accelerator should be avoided since this significantly shortens the setting time.

#### 3.1.3. Effect of the Retarder and Accelerator

**Figure 10:**
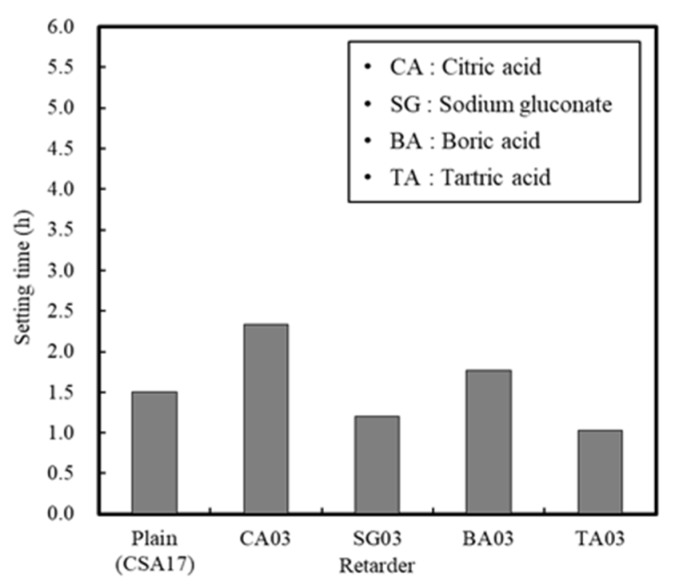
Mortar setting times in the presence of different retarders.

The variation in mortar setting time in the presence of different retarders is outlined in Figure 10. As indicated, CA exhibited the greatest retardation effect, followed by BA, SC, and TA. Indeed, CA and SG are widely used as retarders for mortar and concrete owing to their excellent economic values and retardation performances. Both CA and SG consist of six carbon atoms although CA contains one –OH group and three –COOH groups, whereas SG contains five –OH groups and one –COOH group. These differences therefore account for their varied performances in the retardation of cement hydration. It should be noted that use of an equivalent amount of CA (i.e., with a linear molecular structure) exhibited the optimal retardation effect.

**Figure 11:**
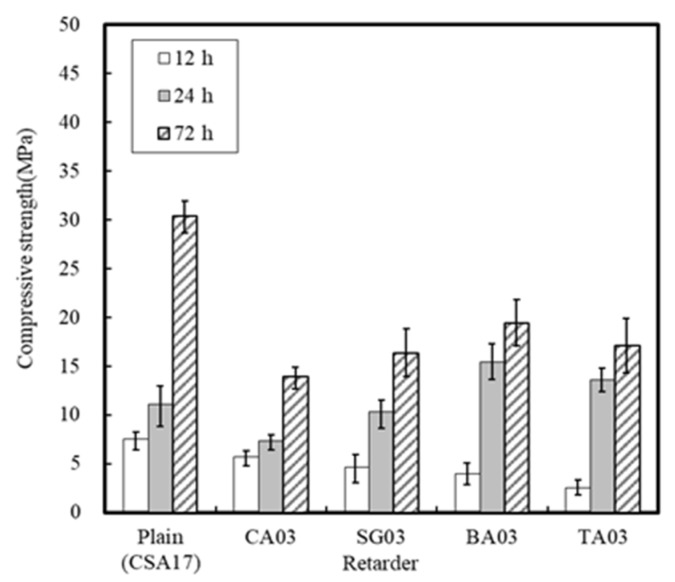
Differences in mortar compressive strengths in the presence of different retarders.

In addition, in the case of the mortar setting time, with the exception of the case of CA, setting took place within 2 h, and so the addition of CA is necessary to secure sufficient constructability. However, Figure 11 shows that the compressive strength of the concrete using the retarder was lowered. It may therefore be possible to accelerate early strength development through the combined use of different accelerators.

**Figure 12:**
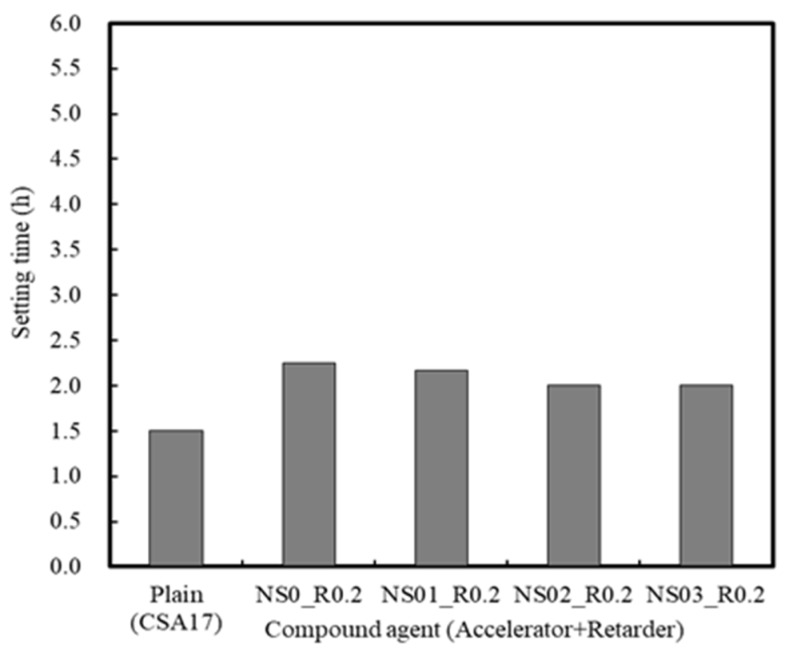
Mortar setting times in the presence of accelerators and retarders.

**Figure 13:**
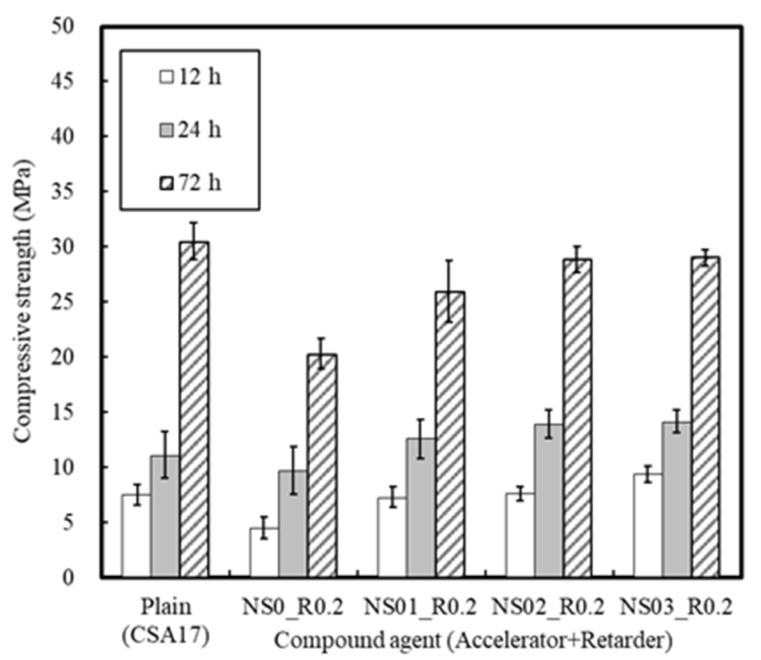
Differences in mortar compressive strengths in the presence of accelerators and retarders.

The variation in mortar setting time and compressive strength in the presence of both an accelerator (A) and a retarder (R) are depicted in Figure 12 and Figure 13. As indicated, the setting time of Plain R0A0 was 60 min, while that of R0.2A0 containing 0.2% retarder was 135 min, thereby indicating a delay in hydration. Consequently, the early compressive strength of the mortar sample decreased significantly to 57% of that of the Plain sample [42].

Compared to the Plain sample (CSA17), in the NS03_R0.2 series, where Na_2_SO_4_ was used as the accelerator, the mortar setting time decreased slightly, but the strength increased steadily within the range of 12–24 h, giving values of 191.1–218.9%.

**Figure 14:**
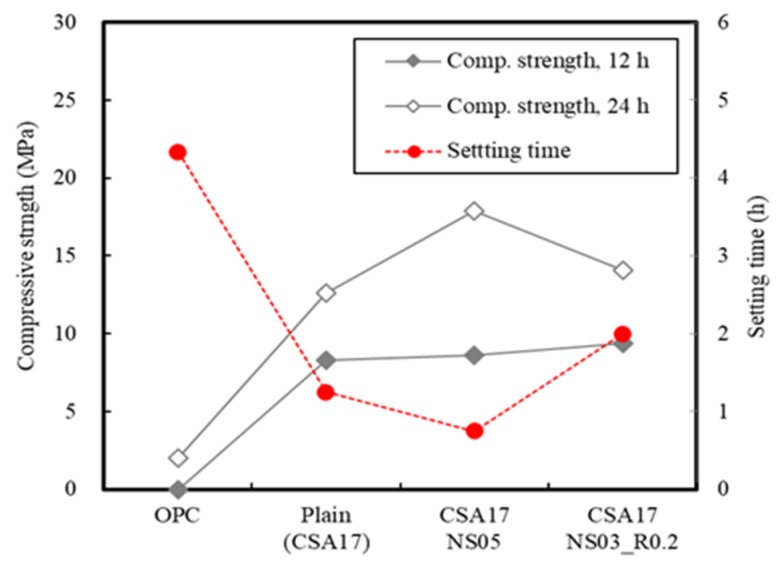
Mortar properties in the presence of CSA and an admixture.

Figure 14 summarizes the setting time and compressive strength results for the mortar samples. More specifically, when 17% CSA was added to the cement, it was evident that the compressive strength of mortar was improved, while the setting time decreased. In addition, the early strength of mortar CSA-containing was increased, but the rate of strength development decreased at 24 h, which indicates that an accelerator is required to secure sufficient mortar strength. Furthermore, when mortar is mixed with Na_2_SO_4_ as an accelerator, early strength development was successful, but the setting time decreased. From this, it was considered that the use of an additional retarder is necessary to secure a stable working time.

Furthermore, the use of retarders in CSA-based mortars has been reported to be an effective approach to improve the workability of a cement blend system that cures rather quickly without an ultimate strength reduction [20,43].

### 3.2. Concrete Properties

**Figure 15:**
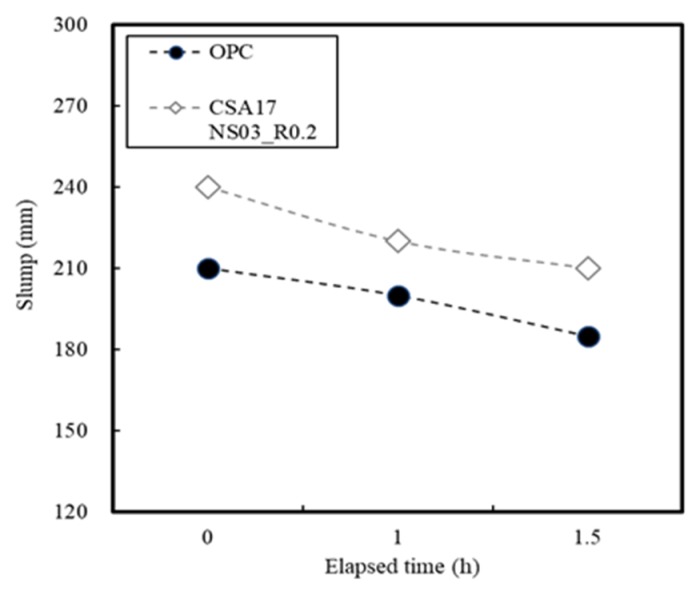
Slump changes in the concrete sample with time.

Figure 15 shows the variation in slump change of the concrete sample with time, where times of 60 and 90 min were examined considering the time between production and arrival onsite. For the purpose of this test, the concrete was rotated at a low speed in the mixer truck, and the slump change was measured at each time point. It was found that OPC and CSA17_NS03_R0.2 led to slightly decreased slumps, but the obtained values remained within the slump range set in the present study, thereby indicating no issues in terms of field workability. From this result, the use of a CSA binder can be used to control the setting properties.

**Figure 16:**
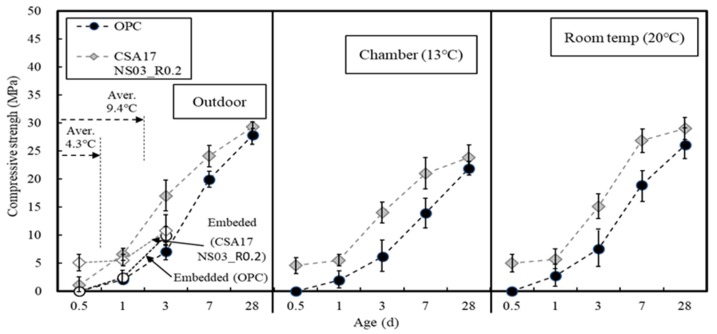
Variation in the compressive strength of concrete with a curing temperature.

Figure 16 shows the variation in the compressive strength of concrete based on the curing temperature. More specifically, in the case of OPC, measurement of the compressive strength of concrete at 12 h was not possible due to the setting and hardening delay under the external curing conditions. After 3 days, an early strength of ≥5 MPa had developed, and upon increasing the temperature, the time taken to develop this degree of early strength tended to decrease. However, the target strength was developed after only 24 h, even at 20 °C.

In the case of the CSA17_NS03_R0.2 sample, a compressive strength of 5 MPa had developed after 24 h at a low temperature <10 °C, and after 12 h above 13 °C. It should be noted here that early-strength-type materials develop an excellent concrete early strength due to the rapid initial reaction but tend to exhibit a delay in long-term strength development. Moreover, the combination of early strength-type materials used in this study was found to result in an excellent strength development for the concrete samples even after 28 days.

**Figure 17:**
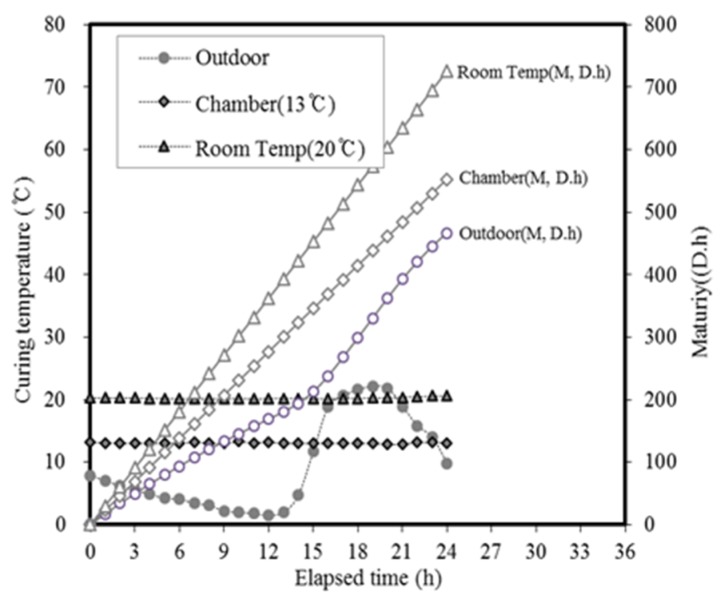
Temperature history and maturity based on the curing method.

**Figure 18:**
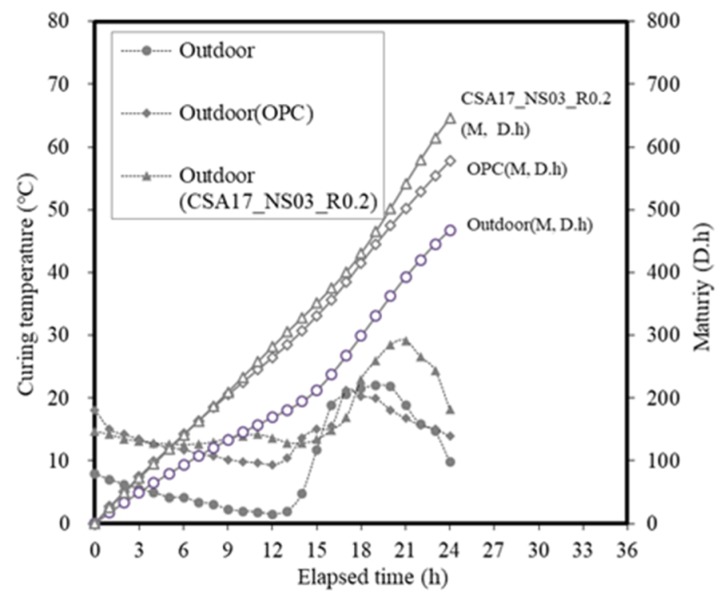
Temperature history and maturity of the concrete samples in the mock-up experiment.

The temperature history and maturity [44] of the curing method are depicted in Figure 17 and Figure 18, respectively. In these experiments, the concrete mixture was cured by maintaining constant temperatures of 13 and 20 °C using constant temperature chambers, and the experimental setup was exposed to outdoor air. During the mock-up experiment, the external temperature was gradually decreased from its initial value of 7.9 °C, and the average temperature values at 12 and 24 h were 4.3 and 9.4 °C, respectively.

**Figure 19:**
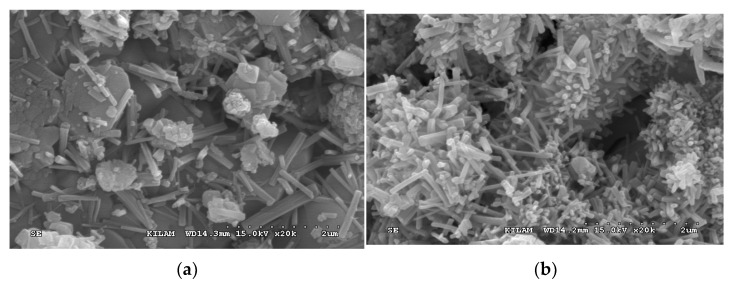
Scanning electron microscopy images of the (**a**) Plain OPC and (**b**) CSA17_NS03_R0.2 samples at 12 h and with a curing temperature of 13 °C.

Overall, the mock-up experiment revealed that the use of CSA17_NS03_R0.2 was advantageous from the viewpoint of early strength development owing to its higher hydration heat and maturity compared to OPC. Figure 19 shows the scanning electron micrographs of OPC and CSA17_NS03_R0.2 captured at 12 h after curing at 13 °C. As can be seen from these images, in the case of CSA17_NS03_R0.2, the hydration product ettringite was produced and activated. However, this was not observed for the Plain OPC sample.

## 4. Conclusions

In this study, the early strength development of concrete based on ordinary Portland cement (OPC) and calcium sulfoaluminate (CSA) cement blends at low temperature curing was examined. The setting time and initial compressive strength of mortar were evaluated by adjusting the amount of CSA-containing OPC, and the optimal performance was observed when using 17% of CSA with respect to the total binder, in addition to CaO/SO_3_ and SO_3_/Al_2_O_3_ ratios of 1.9 and 1.25, respectively. Furthermore, the effect of the accelerator in the development of the mortar early compressive strength decreased in the order of NS > AS > CH > NC > CN ≥ Plain. The setting time of the Plain mortar without the addition of an accelerator was 90 min, whereas this time rapidly decreased to 20–40 min in the presence of an accelerator, suggesting that the using of an accelerator alone is not desirable. Moreover, setting experiments using CSA binders demonstrated that the most effective retardation effect was in the order of citric acid > boric acid > sodium gluconate > tartaric acid. Setting was retarded at a maximum of 141 min using citric acid, as compared to the Plain sample (CSA17). A combination of accelerator and retarder was effective in achieving early strength for the mortar sample containing Na_2_SO_4_ as an accelerator. However, the setting time was shortened, and so an additional retarder should be used to secure sufficient working time. In addition, upon combination of the concrete with an early strength binder (i.e., CSA, accelerator, and retarder), little slump change was observed over 90 min, and a compressive strength >5 MPa/12 h was confirmed following the initial hydration under low temperature conditions. Finally, although the early strength development of concrete was observed in this study at low temperatures, the fine concrete combination adjustment and performance of early-strength binders under different operating conditions must be examined to facilitate the attainment of a qualitatively stable concrete mixture.

## Figures and Tables

**Table 1:** Chemical compositions of the binders employed.

Materials	Chemical Compositions (%)									
	CaO	SiO_2_	Al_2_O_3_	Fe_2_O_3_	MgO	K_2_O	Na_2_O	TiO_2_	SO_3_	LOI
OPC ^(1)^	60.2	21.6	5.15	3.30	2.30	0.99	0.53	-	1.50	2.41
GGBS ^(2)^	44.9	35.4	13.0	0.47	5.01	0.37	-	-	1.31	0.69
FA ^(3)^	4.00	57.9	20.5	6.80	1.38	1.18	0.89	0.13	-	4.60
CSA ^(4)^	45.51	4.91	22.36	1.74	1.57	0.17	0.43	-	22.63	1.90

(1) OPC: ordinary Portland cement; (2) GGBS: ground granulated blast-furnace slag; (3) FA: fly ash; (4) CSA: calcium sulphoaluminate.

**Table 2:** Physical properties of the materials employed.

Material		Property
OPC		ASTM Type I Ordinary Portland cement Density: 3150 kg/m^3^, fineness: 330 m^2^/kg
FA		Fly Ash Density: 2140 kg/m^3^, fineness: 396 m^2^/kg
GGBS		Ground granulated blast-furnace Slag Density: 2860 kg/m^3^, fineness: 430 m^2^/kg
CSA		Calcium sulphoaluminate Density: 2890 kg/m^3^, fineness: 466 m^2^/kg
Fine aggregate	S1	ISO Standard sand, Size: 2 mm Fineness modulus: 2.99, density: 2620 kg/m^3^, SiO_2_: 99%
	S2	Sea sand, Size: 5 mm Fineness modulus: 2.01, density: 2600 kg/m^3^, absorption: 0.79%
	S3	Crushed sand, Size: 5 mm Fineness modulus: 3.29, density: 2570 kg/m^3^, absorption: 0.87%
Coarse aggregate		Crushed granitic aggregate, Size: 25 mm Fineness modulus: 6.94, density: 2600 kg/m^3^, absorption: 0.76%
Chemical admixture		Polycarboxylic acid group, density: 1260 kg/m^3^
Accelerator material	NS	Na_2_SO_4_, density: 3350 kg/m^3^, solubility: 13.9 g/100 mL (20 °C)
	AS	Al_2_(SO_4_)_3_, density: 2672 kg/m^3^, solubility: 36.4 g/100 mL (20 °C)
	CH	Ca(OH)_2_, density: 2211 kg/m^3^, solubility: 17.3 g/100 mL (20 °C)
	CN	Ca(NO_3_)_2_, density: 2504 kg/m^3^, solubility: 51.4 g/100 mL (20 °C)
	NC	NaHCO_3_, density: 2200 kg/m^3^, solubility: 9.6 g/100 mL (20 °C)
Retarder		CA: Citric acid, SG: Sodium gluconate, BA: Boric acid, TA: Tartaric acid

**Table 3:** Experimental outline.

Series	Type	Factor	CSA Rate of OPC (%)	Curing Temperature (°C)	Chemical Admixture	Evaluation Item
Ⅰ	Mortar	Replacement ratio of CSA	0, 13, 14, 15, 16, 17, 18	20	-	Setting time (h) Compressive strength -12 h, 24 h
		Accelerator	17	20	NS, AS, CH, CN, NC (B × 0.5%)	Setting time (h) Compressive strength -12 h, 24 h, 72 h
		Retarder	17	20	CA, SG, BA, TA (B × 0.2%)	
		Accelerator + Retarder	17	20	NS (B × 1%, 2%, 3%) CA (B × 0.2%)	
Ⅱ	Concrete	Application (Batch plant + Mock up member)	17	Outdoor Air Chamber (13) Room temp(20)	AD+ NS + CA (B × 3.2)	Slump Compressive strength-12 h, 24 h, 72 h 7 D, 28 D-Mock up member: 12 h, 24 h, 72 h

**Table 4:** Mixing proportions of basic mortar.

Series	W/C (%)	C:S ^(1)^	Cement (g)	Water (g)	AD ^(2)^ (B ×%)
Ⅰ (Mortar)	50	1:3	450	225	0.7

(1) C:S = Cement: Sand, S: ISO Standard sand (S1); (2) AD: Admixture.

**Table 5:** Mixing proportions of concrete.

Series		W/B (%)	S/a (%)	Unit Weight (kg/m^3^)							AD ^(3)^ (B ×%)	AC (B ×%)
				W	C ^(1)^	CSA	GGBS	FA	S ^(2)^	G		
Ⅱ (Concrete)	Plain	53.0	49.0	175	215	-	66	50	880	916	0.7	-
	CA17	53.0	49.0	175	274	56	-	-	899	901	-	3.1

(1) C: ordinary Portland cement, CSA: calcium sulphoaluminate, GGBS: ground granulated blast-furnace Slag, FA: fly ash; (2) S: Sea sand (S2) + Crushed sand (S3); (3) AD: Admixture, AC: Admixture + Accelerator + Retarder.

**Table 6:** Test methods and their corresponding evaluation items.

Series	Evaluation Item	Test Method	Size (mm)
Ⅰ. Mortar test	Setting time (h)	ASTM C403/C403M [33]	-
	Compressive strength (MPa)	ASTM C109/C109M [34]	40 × 40 × 160
Ⅱ. Concrete test	Slump (mm)	ASTM C143 [35]	-
	Air content (%)	ASTM C231 [36]	-
	Compressive strength (MPa)	ASTM C873 [37]	Ø100 × 200
		ASTM C39 [38]	
